# Deep Learning-Based Functional Independence Measure Score Prediction After Stroke in Kaifukuki (Convalescent) Rehabilitation Ward Annexed to Acute Care Hospital

**DOI:** 10.7759/cureus.16588

**Published:** 2021-07-23

**Authors:** Masahito Katsuki, Norio Narita, Dan Ozaki, Yoshimichi Sato, Wenting Jia, Taketo Nishizawa, Ryuzaburo Kochi, Kanako Sato, Kokoro Kawamura, Naoya Ishida, Ohmi Watanabe, Siqi Cai, Shinya Shimabukuro, Iori Yasuda, Kengo Kinjo, Kenichi Yokota

**Affiliations:** 1 Neurosurgery, Kesennuma City Hospital, Kesennuma, JPN; 2 Neurosurgery, Itoigawa General Hospital, Itoigawa, JPN

**Keywords:** artificial intelligence (ai), deep learning (dl), functional independent measure (fim), kaifukuki (convalescent) rehabilitation ward (krw), stroke, prediction model, prediction one, sony network communications inc. japan

## Abstract

Introduction

Prediction models of functional independent measure (FIM) score after kaifukuki (convalescent) rehabilitation ward (KRW) are needed to decide the treatment strategies and save medical resources. Statistical models were reported, but their accuracies were not satisfactory. We made such prediction models using the deep learning (DL) framework, Prediction One (Sony Network Communications Inc., Tokyo, Japan).

Methods

Of the 559 consecutive stroke patients, 122 patients were transferred to our KRW. We divided our 122 patients’ data randomly into halves of training and validation datasets. Prediction One made three prediction models from the training dataset using (1) variables at the acute care ward admission, (2) those at the KRW admission, and (3) those combined (1) and (2). The models’ determination coefficients (R^2^), correlation coefficients (rs), and residuals were calculated using the validation dataset.

Results

Of the 122 patients, the median age was 71, length of stay (LOS) in acute care ward 23 (17-30) days, LOS in KRW 53 days, total FIM scores at the admission of KRW 85, those at discharge 108. The mean FIM gain and FIM efficiency were 19 and 0.417. All patients were discharged home. Model (1), (2), and (3)’s R^2^ were 0.794, 0.970, and 0.972. Their mean residuals between the predicted and actual total FIM scores were -1.56±24.6, -4.49±17.1, and -2.69±15.7.

Conclusion

Our FIM gain and efficiency were better than national averages of FIM gain 17.1 and FIM efficiency 0.187. We made DL-based total FIM score prediction models, and their accuracies were superior to those of previous statistically calculated ones. The DL-based FIM score prediction models would save medical costs and perform efficient stroke and rehabilitation medicine.

## Introduction

In Japan, stroke is the third leading cause of death and the second leading cause of long-term disability. Japan started a characteristic rehabilitation system in 2000 called kaifukuki (convalescent) rehabilitation wards (KRWs). KRW is incorporated into the Japanese medical insurance system, and The Japan Ministry of Health, Labour, and Welfare define KRWs as the essential inpatient rehabilitation system. Stroke patients are eligible for the KRW. They can undergo rehabilitation up to 150 days and 3 hours per day of rehabilitation, including physical, occupational, and speech therapy, in the KRW. There are over 60 KRW beds per 100,000 individuals, comprising 4.6% of the total Japanese hospital beds, and the number of KRW is increasing [1].

According to the Japanese Guidelines for the Management of Stroke 2015, rehabilitation should be performed to predict functional prognosis, hospital stay, and outcome-based activities of daily living, functional disability, and comorbidities [2]. Also, the KRW’s quality should be maintained, and we should save the limited medical resources. Since 2017, the functional independence measure (FIM) score [3] has been used as a quality indicator of KRW, and hospitals with KRWs must achieve certain FIM-related standards [1]. Therefore, we try to increase and predict the total FIM score, FIM gain, and FIM efficiency (FIM gain divided by length of stay (LOS) in KRW) of each patient. Previously, several reports used multiple regression to predict total FIM score [4-15], but there are some questionable points; (1) not doing validation, (2) not confirming the normal distribution of variables and residuals to use multiple regression [16].

Generally, statistically making a prediction model needs many samples over thousands, so these studies tend to be country-initiated or academic association-initiated research. However, the larger the sample size, the less detailed information is available, such as comorbidities or laboratory test data, and the more there are missing data. Also, the treatment strategies vary from hospital to hospital, and patient backgrounds differ depending on countries and regions. Therefore, statistically making a universal prediction model for the FIM score is very difficult [16].

Recently, deep learning (DL), contained in artificial intelligence, is attractive. DL is gradually starting to be used in neurosurgical diseases in decision making for spinal canal stenosis [17], predicting outcomes after stroke [18,19], predicting the occurrence of stroke from meteorological information [20], automated diagnosis of primary headaches from a Japanese medical questionnaire [21], pathological diagnosis [22], or radiomics studies of brain tumours [23]. However, there are no reports on the DL-based prediction model regarding the total FIM score after KRW admission. We hypothesized that we could make a good prediction model for our hospital using the DL framework, even with a small dataset. Therefore, we herein produced the prediction model using DL framework, Prediction One (Sony Network Communications Inc., Tokyo, Japan, https://predictionone.sony.biz/) [19-21,24,25] with our dataset and compared the utility of the DL-based model to previously-reported multiple regression models. We also investigated our patients’ characteristics admitted to KRW because our hospital is unique in that KRW is annexed to an acute care hospital.

## Materials and methods

Study population

We retrospectively retrieved data from medical records of all the consecutive 559 stroke patients admitted between 2017 and 2019 and treated at our institution. The stroke diagnosis was based on the clinical history and the findings of computed tomography (CT) or magnetic resonance imaging. 

General management of stroke, including cerebral infarction (CI), intracerebral haemorrhage (ICH), and subarachnoid haemorrhage (SAH), was according to the Japanese Guidelines for the Management of Stroke 2015 [2]. We first performed acute care, including appropriate medical treatment and surgery. Rehabilitation is limited to 60 min in the acute phase. After 1-2 weeks of acute-phase rehabilitation, we discussed the patients’ final destination with their families; home, KRW, nursing facilities, or long term hospitals. Patients who could live independently or with sufficient support from their families were discharged home in 2-4 weeks. Patients who needed more rehabilitation and might have the potential to live independently or with families’ supports could be transferred to KRW. Patients who would not live independently or bed-ridden but whose families committed to providing in-home care could also be transferred to KRW, and such patients must have been discharged home. Patients who needed care and whose families could not support them were discharged to nursing facilities. Patients with severe neurological deficits like vegetative states were transferred to long term hospitals.

In the KRW, we could provide rehabilitation that was in line with real life. The daily rehabilitation was up to 3 hours, and the LOS was up to 150 days as maximum. We, doctors, nurses, and therapists, held monthly meetings with the patients and their families together, and we decided how long the rehabilitation in the KRW would be. This study retrospectively investigated the patients’ characteristics, including age, stroke types, and discharge destination.

Clinical variables for making prediction models

We collected data regarding physiological symptoms at admission, i.e., date of admission, age, sex, height, weight, body mass index, history of smoking and habitual massive drinking (over 450g ethanol intake/week), comorbidities (history or present treatment by a clinician for hypertension, dyslipidemia, diabetes mellitus, chronic kidney diseases, orthopaedic diseases, or cancer). Glasgow Coma Scale (GCS) and National Institutes of Health Stroke Scale scores on admission were also investigated. We also measured triglycerides, total cholesterol, high-density lipoprotein cholesterol, low-density lipoprotein cholesterol, albumin, C-reactive protein, blood glucose, haemoglobin A1c, haemoglobin levels, and white blood cell and lymphocyte counts admission in the acute neurosurgical ward. Albumin, lymphocyte, and total cholesterol are known factors for controlling nutritional status scores to assess the patients’ nutritional status [26]. As radiological findings, we investigated temporal muscle thickness (TMT) (mm) as an indicator of systemic skeletal muscle mass [27-34] based on the results of the CT at admission. SYNAPSE V 4.1.5 imaging software (Fujifilm Medical, Tokyo, Japan) was used through the methods described previously [32,33].

We also investigated the modified Rankin Scale scores when the patients were transferred to the KRW. The LOS in the acute care ward and KRW were also investigated. Barthel Index (BI) at the admission of acute care ward, FIM score at admission to KRW and the discharge of KRW, FIM gain, and FIM efficiency were also collected, and the total FIM score at discharge was defined as an outcome in this study. There were no missing values.

Making prediction model by Prediction One

We used Prediction One framework to make the prediction models. We divided our 122 patients’ data randomly into 61 patients training dataset and 61 patients validation dataset. Prediction One read the training data and automatically performed 5-folds cross-validation. Prediction One automatically adjusted and optimized the easy to process variables statistically and mathematically and select appropriate algorithms with ensemble learning. Prediction One made the best prediction model by an artificial neural network with internal cross-validation. The details are trade secrets and could not be provided.

We let the Prediction One framework make three prediction models using training dataset; (1) using 49 variables acquired only at the admission of acute care ward, (2) using 40 variables, including FIM scores, which could be known only at the admission of KRW, and (3) using all 70 variables acquired both at the admissions of acute care and KRW. The determination coefficient (R^2^) and strong variables of each model were automatically calculated. Then, we performed validation using the validation datasets. Correlation coefficients (r) and residuals between the predicted and actual total FIM scores were calculated to evaluate the models’ accuracy.

Statistical analysis

Results are basically shown as median (interquartile range). The difference between the training dataset and the external validation dataset was tested appropriately using the Mann-Whitney U test, Fisher’s exact test, or Pearson’s chi-square test. Univariate analysis on the association between each variable and total FIM score at the discharge of KRW was also performed. We could not perform multiple regression due to the small sample size and non-normal distribution of variables. A two-tailed p < 0.05 was considered statistically significant. We calculated r and these p values using SPSS software version 24.0.0. (IBM, New York, USA).

## Results

Clinical characteristics

The clinical characteristics of the 559 stroke patients (421 CI, 98 ICH, 40 SAH; 224 women and 335 men) are summarized in table 1. The median (interquartile range) age was 77 (69-83), and LOS in the acute care ward 15 (7-26). Regarding discharge destinations, 205 patients were discharged home, 122 patients were transferred to KRW, 81 nursing facilities, 79 long-term hospitals, and 72 patients died.

**Table 1:** Characteristics of all stroke patients KRW; kaifukuki (convalescent) rehabilitation ward, LOS; length of stay.

	All stroke (n = 559)	CI (n = 421, 75%)	ICH (n = 98, 18%)	SAH (n = 40, 7%)
Age (median, interquartile range)	77 (69-83)	77 (70-83)	79 (69-83)	66 (59-83)
32-50 y.o.	17 (3%)	10 (2%)	5 (5%)	2 (5%)
51-65 y.o.	68 (12%)	39 (9%)	13 (13%)	16 (40%)
66-75 y.o.	165 (30%)	139 (33%)	17 (18%)	9 (21%)
76-85 y.o.	224 (40%)	163 (39%)	51 (52%)	11 (27%)
86-95 y.o.	79 (14%)	66 (16%)	12 (12%)	1 (2%)
95- y.o.	6 (1%)	4 (1%)	0	2 (5%)
Women: Men (Women%)	224: 335 (40%)	167: 254 (40%)	36: 62 (37%)	21: 19 (53%)
Discharge destination from the acute ward				
Home	205 (37%)	162 (38%)	32 (33%)	11 (27%)
KRW	122 (22%)	73 (17%)	33 (33%)	17 (43%)
Nursing facility	81 (14%)	62 (15%)	17 (17%)	2 (5%)
Long term hospital	79 (14%)	64 (16%)	13 (13%)	2 (5%)
Dead	72 (13%)	60 (14%)	4 (4%)	8 (20%)
LOS in an acute ward (days) (median, interquartile range)	15 (7-26)	19 (7-45)	21 (12-44)	10 (1-39)

Table 2 shows the treatment detail of 122 patients (67 CI, 42 ICH, and 13 SAH) who were transferred to KRW. Thirty-nine (32%) of the 122 patients underwent aggressive treatment like intravenous tissue plasminogen activator, thrombectomy, hematoma removal surgery, or aneurysm ligation, rather than only medication.

**Table 2:** Stroke types and treatment of patients who underwent KRW ACoA; anterior communicating artery, ACD; distal part of anterior cerebral artery, A-to-A; artery to artery embolism, ATBI; atherothrombotic brain infarction, BA; basilar artery, BAD; brunch atheromatous disease, CE; cardiogenic embolism, CI; cerebral infarction, ESUS; embolic stroke of undetermined sources, ICA; internal carotid artery, ICH; intracerebral hemorrhage, LI; lacunar infarction, MCA; middle cerebral artery, SAH; subarachnoid hemorrhage, t-PA; tissue-plasminogen activator; VAD; vertebral artery dissection.

	Number		Number		Number
CI	67 (5 t-PA, 5 t-PA + thrombectomy, 2 thrombectomy, 2 craniectomy)	ICH	42 (9 surgery)	SAH	13
ATBI	16 (3 t-PA, 1 craniectomy)	Putamen	16 (5 surgery)	ACoA (clip)	3
LI	11 (1 t-PA)	Thalamus	8	ACD (clip)	1
CE	16 (5 t-PA + thrombectomy, 2 thrombectomy, 1 craniectomy)	Subcortex	8 (3 surgery)	MCA (clip)	4
BAD	18	Pons	3	ICA (clip)	3
VAD	3	Cerebellum	7 (1 surgery)	BA (coil)	1
ESUS	2 (1 t-PA)			VAD (coil)	1
A-to-A	1				

Table 3 shows the characteristics of the 122 patients (49 women and 73 men). The median age was 71 (64-79), LOS in acute care ward 23 (17-30) days, LOS in KRW 53 (35-92) days, total FIM scores at admission of KRW 85 (57-100), those at discharge 108 (86-118), FIM gain 19 (8-89), and FIM efficiency 0.271 (0.147-0.561). All 122 patients were discharged home. The mean FIM gain and FIM efficiency were 19 and 0.417, although they were not with a normal distribution (Table 3). Age (r = -0.487, p < 0.001), LOS in KRW (r = -0.501, p < 0.001), body weight (r = 0.322, p < 0.001), GCS score at admission (r = 0.350, p < 0.001), BI at admission of acute care ward (r =0.435, p < 0.001), total FIM score at admission of KRW (r = 0.874, p < 0.001) were significantly associated with total FIM score at discharge of KRW in univariate analyses.

**Table 3:** Detailed characteristics of patients who were hospitalized in KRW CI; cerebral infarction, FIM; functional independent measure, ICH; intracerebral hemorrhage, KRW; kaifukuki (convalescent) rehabilitation ward, LOS; length of stay, NIHSS; National Institutes of Health Stroke Scale, SAH; subarachnoid hemorrhage, SD; standard deviation.

	Total (n = 122)		CI (n = 67)		ICH (n = 42)		SAH (n = 13)	
Age (median, interquartile range)	71 (64-79)		53 (35-92)		70 (65-78)		65 (57-75)	
Women: Men (Women%)	49:73		24:43		19:23		6:7	
Body mass index (kg/m^2^)	22.5 (20.8-25.0)		22.5 (20.8-25)		22.3 (19.6-24.4)		22.8 (21.1-24.5)	
Past history								
Presence of hypertension	89 (73%)		46 (69%)		33 (76%)		10 (77%)	
Presence of diabetes mellitus	29 (24%)		19 (28%)		9 (21%)		1 (8%)	
Presence of sydlipidemia	30 (26%)		17 (25%)		7 (17%)		6 (46%)	
History of smoking	62 (51%)		35 (52%)		20 (48%)		7 (54%)	
History of massive alcohol comsumption	35 (29%)		21 (31%)		10 (24%)		4 (31%)	
Presence of chronic kidney disease	10 (8%)		5 (7%)		4 (10%)		1 (8%)	
Presence of orthopedic disease	15 (12%)		8 (12%)		6 (14%)		1 (8%)	
Presence of malignant tumor	7 (6%)		5 (7%)		0		2 (15%)	
Glasgow Coma Scale on admission	14 (14-15)		15 (14-15)		14 (13-14)		14 (10-15)	
NIHSS on admission	5 (3-11)		5 (3-10)		8 (3-15)		-	
Barthel index on admission	23 (0-41)		25 (5-45)		5 (0-30)		5 (0-35)	
Laboratory test								
Triglycerides (mg/dL)	99 (68-144)		105 (73-148)		88 (65-143)		96 (81-127)	
Total cholesterol (mg/dL)	191 (169-222)		186 (168-214)		199 (166-229)		203 (186-223)	
High-density lipoprotein cholesterol (mg/dL)	49.9 (42.5-68.9)		48.1 (42.2-64.0)		48.6 (45.5-75.0)		71.0 (48.1-75.0)	
Low-density lipoprotein cholesterol (mg/dL)	114 (93-137)		115 (94-137)		113 (93-139)		116 (93-137)	
Albumin (g/dL)	4.3 (4.0-4.5)		4.3 (4.0-4.6)		4.3 (4.0-4.5)		7.3 (6.8-7.5)	
C-reactive protein (mg/dL)	0.2 (0.1-0.4)		0.2 (0.1-0.4)		0.2 (0.1-0.5)		0.2 (0.1-0.3)	
Blood glucose (mg/dL)	133 (108-165)		130 (106-168)		133 (112-159)		144 (122-163)	
HbA1c (%)	5.8 (5.5-6.4)		5.9 (5.5-6.6)		5.9 (5.4-6.3)		5.5 (5.4-5.7)	
White blood cell count (/μL)	7400 (6000-9900)		7150 (5750-8800)		7750 (6000-10400)		8800 (7200-10300)	
Hemoglobin (g/dL)	13.8 (12.6-15.4)		13.8 (12.6-15.9)		13.5 (12.4-15.2)		13.9 (13.3-15.3)	
Lymphocyte count (/μL)	1611 (1198-2159)		1570 (1185-2025)		1540 (1309-2240)		2083 (1627-3313)	
Temporal muscle thickness (mm)	4.6 (3.6-5.9)		4.5 (3.7-5.7)		4.8 (3.7-5.9)		5.0 (3.5-6.8)	
Modified Rankin Scale when transferred	3 (3-4)		3 (3-4)		4 (3-5)		2 (2-4)	
LOS in an acute ward (days) (median, interquartile range)	23 (17-30)		23 (17-29)		22 (17-29)		30 (26-33)	
FIM scores (median, interquartile range)	admission to KRW	discharge of KRW	admission to KRW	discharge of KRW	admission to KRW	discharge of KRW	admission to KRW	discharge of KRW
Self-care								
Eating	6 (5-7)	7 (5-7)	6 (5-7)	7 (5-7)	5 (4-6)	6 (5-7)	7 (5-7)	7 (7-7)
Grooming	5 (4-7)	7 (5-7)	5 (4-7)	7 (5-7)	5 (3-5)	6 (5-7)	7 (4-7)	7 (7-7)
Bathing	4 (1-5)	5 (4-7)	4 (1-5)	5 (5-7)	2 (1-4)	5 (3-5)	3 (1-6)	7 (5-7)
Dressing-Upper body	5 (2-7)	7 (5-7)	5 (4-7)	7 (6-7)	4 (2-6)	6 (4-7)	6 (3-7)	7 (7-7)
Dressing-Lower body	5 (1-7)	6 (5-7)	5 (4-7)	7 (6-7)	4 (1-5)	6 (4-7)	5 (3-7)	7 (7-7)
Toileting	6 (2-6)	6 (6-7)	6 (4-7)	7 (5-7)	4 (1-6)	6 (4-7)	7 (5-7)	7 (6-7)
Sphincter Control								
Bladder Management	5 (2-7)	7 (5-7)	7(4-7)	6 (5-7)	4 (1-6)	6 (4-7)	7 (3-7)	7 (7-7)
Bowel Management	5 (4-6)	5 (5-7)	5 (5-6)	6 (5-7)	4 (2-6)	5 (5-6)	5 (4-5)	5 (5-6)
Transfer								
Bed, Chair, Wheelchair	5 (3-7)	6 (5-7)	5 (5-7)	6 (6-7)	5 (2-6)	5 (5-7)	7 (4-7)	7 (7-7)
Toileting	5 (3-6)	6 (5-7)	6 (5-7)	5 (4-7)	4 (2-5)	6 (5-7)	7 (4-7)	7 (7-7)
Tub, Shower	1 (1-5)	5 (1-6)	1 (1-5)	6 (5-7)	5 (1-6)	4 (1-5)	1 (1-2)	6 (5-6)
Locomotion								
Walk/Wheelchair	5 (1-6)	6 (5-7)	5 (4-6)	6 (5-7)	1 (1-5)	5 (4-7)	5 (4-7)	7( 7-7)
Stairs	1 (1-5)	5 (4-6)	1 (1-5)	5 (5-6)	1 (1-1)	5 (3-5)	3 (1-5)	5 (5-6)
Motor Subtotal Score	62 (36-72)	80 (62-87)	66 (51-75)	81 (67-87)	45 (22-65)	73 (55-81)	69 (47-77)	87 (82-88)
Communication								
Comprehension	5 (3-6)	6 (5-7)	5 (4-6)	6 (5-7)	5 (3-6)	6 (5-7)	5 (2-7)	7 (5-7)
Expression	5 (3-6)	6 (5-7)	5 (4-6)	7 (5-7)	4 (3-6)	6 (5-7)	4 (3-7)	7 (5-7)
Social Cognition								
Social Interaction	6 (4-6)	7 (6-7)	6 (5-6)	7 (6-7)	5 (4-6)	6 (6-7)	5 (3-6)	7 (6-7)
Problem Solving	4 (2-5)	5 (3-6)	5 (2-5)	5 (4-6)	2 (2-6)	5 (2-5)	5 (2-5)	5 (5-7)
Memory	4 (3-6)	6 (4-7)	5 (3-6)	6 (5-7)	3 (2-5)	5 (4-6)	5 (2-5)	5 (4-6)
Cognitive Subtotal Score	23 (17-29)	29 (23-32)	25 (18-29)	30 (24-32)	20 (14-26)	28 (21-31)	22 (13-30)	30 (26-33)
Total FIM Score	85 (57-100)	108 (86-118)	91 (73-105)	111 (89-120)	70 (44-89)	97 (72-109)	97 (83-103)	
LOS in KRW (days) (median, interquartile range)	53 (35-92)		47 (35-68)		66 (45-117)		36 (32-67)	
FIM gain (median)	19 (8-89)		16 (7-25)		25 (15-31)		16 (12-24)	
FIM efficiency (median)	0.271 (0.147-0.561)		0.286 (0.133-0.589)		0.254 (0.146-0.423)		0.400 (0.105-0.735)	
FIM gain (mean±SD)	19±19		16±11		23±17		16±12	
FIM efficiency (mean±SD)	0.417±0.652		0.456±0.820		0.308±0.280		0.457±0.385	

Model development and validation

There were no significant differences in the variables between the training and validation datasets. Prediction One produced each prediction model in less than two minutes. The R^2^ of each model were described in Table 4. Model (1), using 49 variables acquired only at the acute care ward admission, had an R^2^ of 0.794. Its r and mean ± standard deviation of residuals between the predicted and actual total FIM scores were 0.372 (95%confident interval 0.120-0.578) and -1.56 ± 24.6. Model (2) using 40 variables acquired only at the admission of KRW had R^2^ of 0.970, and its r and residuals were 0.789 (0.788-0.790) and -4.49 ± 17.1. Model (3) using all 70 variables acquired both in the acute and KRW had R^2^ of 0.974, and its r and residuals were 0.810 (0.810-0.811) and -2.69 ± 15.7. The results are compared to those of previous reports [4-15] (Table 4).

**Table 4:** Previous prediction models for total FIM score at discharge FIM; functional independent measure, KRW; kaifukuki (convalescent) rehabilitation ward, LOS; length of stay, r; correlation coefficients, R^2^; determination coefficients

Year	Author	Number of variables	R^2^	institutions	Number of patients	Mean age	Mean LOS in the acute ward (days)	Mean LOS in the KRW (days)	Validation	R^2^ of validation	r of validation	mean residual±SD
1997	Liu [4]	1	0.798	single	106	56.5	83	105.5	-			
2000	Tsuji [5]	3	0.64	multiple	190 training + 116 validation	61	47.3	90.9	+	0.68		
2001	Inouye [6]	1	0.57	single (aged over 80)	464	60	74	116	-			
2001	Inouye [6]	4	0.76	single (aged 60-69)	464	60	74	116	-			
2005	Sonoda [7]	4	Not described	single	87 training + 44 validation	63.4	81.3	154.3	+		0.88	8.06 ± 6.29
2016	Tsuchiya [8]	3	0.30	single	108	66.8	45.5	99.9	-			
2016	Senda [9]	5	0.591	single	520	72.8	32.8	55.4	-			
2016	Nishioka [10]	8	0.663	single	897	71.6	21 (median)	122 (median)	-			
2017	Shiraishi [11]	3	0.776	single	108 (including not only stroke patients)	80.5	Not described	65.2	-			
2019	Matsushita [12]	3	0.673	single	267	72.5	24	105.1	-			
2019	Nishioka [13]	6	0.742	multiple	5549 (including not only stroke patients)	82	Within 2 months.	72	-			
2019	Yoshimura [14]	6	0.719	single	276	74.9	15	99.9	-			
2019	Senda [15]	2	0.691	single	371	72.9	31.4	79.7	-			
2021	Ours	49	0.794	single	61 training + 61 validation	69.4	24.3	53	+		0.372 (0.120-0.578)	-1.56 ± 24.6
2021	Ours	40	0.970	single	61 training + 61 validation	69.4	24.3	53	+		0.789 (0.788-0.790)	-4.49 ± 17.1
2021	Ours	70	0.974	single	61 training + 61 validation	69.4	24.3	53	+		0.810 (0.810-0.811)	-2.69 ± 15.7

The more robust variables of each model are listed in Table 5. In model (1), TMT, lymphocyte count, low-density lipoprotein cholesterol level, total BI score, and haemoglobin level had enormous effects on the outcome. In model (2), Age, FIM (bowel management), TMT, FIM (toileting), and LOS in the acute care ward were important. In model (3), Total FIM scores, subtotal cognitive FIM score, FIM (comprehension), subtotal motor FIM score, and blood glucose levels were meaningful, in order.

**Table 5:** Stronger variables of each model provided by Prediction One (Sony Network Communications Inc., Japan) BI; Barthel index, FIM; functional independent measure, KRW; kaifukuki (convalescent) rehabilitation ward, LOS; length of stay, NIHSS; National Institutes of Health Stroke Scale

Order	Model (1) using 49 variables acquired only at the admission of acute care ward	Model (2) using 40 variables acquired only at the admission of KRW	Model (3) using all 70 variables acquired at the admission of both in acute care and KRWs
1	Temporal muscle thickness	Age	Total FIM scores
2	Lymphocyte	FIM bowel management	Subtotal cognitive FIM score
3	Low-density lipoprotein cholesterol	Temporal muscle thickness	FIM comprehension
4	Total Barthel Index score	FIM toileting	Subtotal motor FIM score
5	Hemoglobin	LOS in the acute ward	Blood glucose
6	NIHSS score	FIM dressing lower body	FIM bathing
7	Blood glucose	FIM expression	Temporal muscle thickness
8	Height	FIM stairs	Total cholesterol
9	Total cholesterol	Height	Total BI at admission
10	BI (on level surfaces)	Subtotal cognitive FIM score	FIM problem solving

## Discussion

We made prediction models for the total FIM score at the discharge of KRW in our hospital. We made three models; (1) using information gained at the admission of acute care ward, (2) using information gained at the admission of KRW, and (3) combined (1) and (2). DL-based models (2) and (3) had good accuracies, and we also performed validation, despite our small dataset. This is the first report on creating DL-based prediction models of total FIM scores at the discharge of KRW.

KRW annexed to acute care hospital

Our hospital was rebuilt in 2017, and KRW was newly annexed. Until then, patients had been transferred to the other hospitals’ KRWs, but it took more than a month from the stroke onset to transfer. Since the new hospital, the transfer to our KRW from the stroke onset has been shortened to 23 days. Furthermore, the mean FIM gain of 19 and the mean FIM efficiency of 0.417 is higher than those of national averages of 17.1 and 0.187 [35]. This may be because the staff in the acute care ward and those in KRW work closely together and frequently held meetings with patients and their families. Sharing the information on patients’ comorbidities, treatment status, and rehabilitation progress will allow us to smooth transfer. This openness is one of the advantages of our hospital.

In our hospital, all patients were discharged home because we only permitted the transfer to KRW for patients who might have the potential to live independently with/without families’ support or those who would not live independently or bed-ridden but whose families committed to providing in-home care. We decided on these determinations after around 1-2 weeks after onset. Whether it is socially and medically right to make such an early decision needs further discussion. However, we are forced to make such early decisions to use our limited medical resources in this rural area effectively. In this situation with limited medical resources, total FIM score prediction at the discharge of KRW is essential for the effective use of limited medical resources and the decision-making of patients and their families.

Advantages of DL

Conventional cost- and time-consuming statistical analysis needs variable optimization like a logarithmic transformation to make the variables with normal distribution increase the prediction model’s accuracy mathematically. It also requires the arbitrary selection of variables based on previous studies, and multivariate analysis needs 10-15 folds number of samples against the variables [36]. Therefore, there is a risk that variables that may be important cannot be used in the statistical analysis. Even the multivariate analysis cannot be done in a small hospital with small data. Furthermore, we should do multiple imputations or listwise deletion in statistical analysis when there is missing data, leading to inaccuracy. Furthermore, we should validate the models’ accuracy, but several previous reports were not validated [16].

DL has the potential to overcome these problems [37]. DL develops useful models with less effort or time using the small dataset, without time-consuming variable optimization nor arbitrarily choosing variables because the DL framework automatically performs these processes. The optimal number of the variables for the DL framework is not revealed, and the DL framework sometimes finds interesting variables as necessary that has not been taken into account in the previously reported statistical models. Furthermore, the DL framework automatically uses appropriate values instead of the missing ones and calculate the best prediction model.

We then review these benefits of DL in our study. Conventionally, we could have used only six variables for statistical analysis due to the small sample size of the training dataset (n = 61). However, we could use 70 variables for making the model (3) and make a good prediction model from the small dataset. We did not need to perform variable optimization by ourselves. Furthermore, some unexpected variables, such as TMT, lymphocyte count, blood glucose level, were judged to be important in DL models. The suggestions are essential because data from the acute phase affects the total FIM score after KRW admission. What was not statistically significant in univariate analyses was deemed essential. Besides, the time needed for creating each model was less than 2 minutes. Finally, the models achieved high accuracies both in the training and validation dataset. While many reports did not conduct validation, we believe that our report is important, creating a stir about validation to present the accuracy.

Limitations of DL

First, the prediction model derived from our data cannot be applied to other institutions. The training and validation dataset must be updated to keep up with advances in medical science and surgical techniques, and medication changes. Creating a DL-based prediction model that can be used universally at any hospital will still require country-initiated or academic association-initiated collaborative research at many institutions. It may eventually require the same amount of effort as the traditional statistical model creation.

Limitation of this study

First, the sample size was small, and it is unknown how many samples are needed for DL analysis. We did not investigate the detail of rehabilitation training and how long the rehabilitation was actually performed per day for each individual. Further continuation study is needed.

## Conclusions

Our hospital has the acute care ward and KRW. The FIM efficiency and LOS in the acute care ward were better than those in national averages. We easily and quickly made total FIM score prediction models using Prediction One framework. The accuracies of the prediction models were superior to those of previous statistically calculated prediction models. Even with a small single-centre dataset, DL-based total FIM score prediction models made by Prediction One can be helpful at the institution. They may be applied to daily clinical practice in the future.
